# A compound heterozygous mutation in HADHB gene causes an axonal Charcot-Marie-tooth disease

**DOI:** 10.1186/1471-2350-14-125

**Published:** 2013-12-05

**Authors:** Young Bin Hong, Ja Hyun Lee, Jin-Mo Park, Yu-Ri Choi, Young Se Hyun, Bo Ram Yoon, Jeong Hyun Yoo, Heasoo Koo, Sung-Chul Jung, Ki Wha Chung, Byung-Ok Choi

**Affiliations:** 1 Department of Neurology, Samsung Medical Center, Sungkyunkwan University School of Medicine, 50 Ilwon-dong Gangnam-Gu, Seoul 135-710, Korea; 2 Department of Biological Science, Kongju National University, 182 Sinkwan-dong, Gongju 314-701, Korea; 3 Department of Neurology, School of Medicine, Ewha Womans University, Seoul, Korea; 4 Department of Biochemistry, School of Medicine, Ewha Womans University, Seoul, Korea; 5 Department of Radiology, School of Medicine, Ewha Womans University, Seoul, Korea; 6 Department of Pathology, School of Medicine, Ewha Womans University, Seoul, Korea

**Keywords:** Charcot-Marie-Tooth disease (CMT), Whole exome sequencing (WES), *HADHB*, Mitochondrial trifunctional protein (MTP)

## Abstract

**Background:**

Charcot-Marie-Tooth disease (CMT) is a heterogeneous disorder of the peripheral nervous system. So far, mutations in hydroxyacyl-CoA dehydrogenase/3-ketoacyl-CoA thiolase/enoyl-CoA hydratase (trifunctional protein), beta subunit (*HADHB*) gene exhibit three distinctive phenotypes: severe neonatal presentation with cardiomyopathy, hepatic form with recurrent hypoketotic hypoglycemia, and later-onset axonal sensory neuropathy with episodic myoglobinuria.

**Methods:**

To identify the causative and characterize clinical features of a Korean family with motor and sensory neuropathies, whole exome study (WES), histopathologic study of distal sural nerve, and lower limb MRIs were performed.

**Results:**

WES revealed that a compound heterozygous mutation in *HADHB* is the causative of the present patients. The patients exhibited an early-onset axonal sensorimotor neuropathy without episodic myoglobinuria, and showed typical clinical and electrophysiological features of CMT including predominant distal muscle weakness and atrophy. Histopathologic findings of sural nerve were compatible with an axonal CMT neuropathy. Furthermore, they didn’t exhibit any other symptoms of the previously reported *HADHB* patients.

**Conclusions:**

These data implicate that mutation in *HADHB* gene can also cause early-onset axonal CMT instead of typical manifestations in mitochondrial trifunctional protein (MTP) deficiency. Therefore, this study is the first report of a new subtype of autosomal recessive axonal CMT by a compound heterozygous mutation in *HADHB*, and will expand the clinical and genetic spectrum of *HADHB*.

## Background

Charcot-Marie-Tooth disease (CMT), also called hereditary motor and sensory neuropathy, is a clinically and genetically heterogeneous disorder of the peripheral nervous system [1-3]. CMT is conventionally divided into the demyelinating form (CMT1) and the axonal form (CMT2). Of them, CMT2 has been divided into many subtypes (from CMT2A to CMT2P) according to their distinct clinical symptoms and genetic causes [4-6].

So far, more than 60 causative genes or loci have been reported to be associated with the CMT development (http://www.molgen.ua.ac.be/CMTMutations/Home/Default.cfm). However, large number of CMT patients is waiting for uncovering their specific genetic underlying causes. Thus efficient analysis tools such as whole exome sequencing (WES) are required to accelerate the identification of the underlying genetic causes. Applications of WES to CMT have been proved as to be powerful to identify rare genetic causes from small-sized pedigrees [7-9].

Mitochondrial trifunctional protein (MTP), a constituent of inner membrane of mitochondria, exerts a significant catalytic activity for beta-oxidation cycle, which metabolizes long-chain fatty acyl-CoA [10]. MTP consists of heterooctamer (α4β4) possessing three different enzyme activities: long-chain enoyl-CoA hydrolase (LCEH) and long-chain 3-hydroxy-acyl-CoA dehydrogenase (LCHAD) activities harbored by the α-subunit (HADHA), while β-subunit (HADHB) encode long-chain 3-ketoacyl-CoA thiolase (LCKT) [10]. Deficiency of MTP is an autosomal recessive disorder that exhibits characteristic features of cardiomyopathy, hypoketotic hypoglycemia, metabolic acidosis, sudden infant death, metabolic encephalopathy, liver dysfunction, axonal sensory neuropathy and pigmentary retinopathy [11-13].

Here, we report clinical and pathological features of an autosomal recessive CMT family harboring a novel compound heterozygous *HADHB* mutation, which is identified by WES.

## Methods

### Patients

We enrolled a total of 6 members of a Korean demyelinating CMT family (family ID: FC354) with 2 affected individuals. The CMT phenotype of this family was seemed to be inherited with autosomal recessive mode, since two of three siblings were affected while both parents were unaffected. No CMT patient was identified from close relatives of the family. This study also recruited 500 Korean healthy controls with no familial history of neuromuscular disorders (10-60 years old). All samples were collected from 2011 to 2012. Informed consent was obtained from all participants and from parents of participants younger than 18 years of age according to the protocol approved by the Institutional Review Board for Ewha Womans University, Mokdong Hospital (ECT 11-58-37).

### Clinical and electrophysiological assessments

Patients were evaluated by taking a detailed history including motor and sensory impairments, deep tendon reflexes, and muscle atrophy undertaken by two independent neurologists. Muscle strengths of flexor and extensor muscles were assessed manually using the medical research council (MRC) scale. In order to determine physical disability, we used three scales, a functional disability scale (FDS) [14], and a CMT neuropathy score (CMTNS) [15]. Sensory impairments were assessed in terms of the level and severity of pain, temperature, vibration and position. Nerve conduction studies (NCSs) were carried out with a surface electrode in median, ulnar, peroneal, tibial, and sural nerves as previously described [16].

### MRI studies

Two patients (II-1 and -2) with *HADHB* mutation were studied with MRI of the brain, hip, thigh and lower leg using a 1.5-T system (Siemens Vision, Siemens, Germany). Whole brains were scanned using a slice thickness of 7 mm and a 2-mm interslice gap, to produce 16 axial images. The imaging protocol consisted of T2-weighted spin echo (TR/TE = 4,700/120 ms), T1-weighted spin echo (TR/TE = 550/12 ms), and fluid-attenuated inversion recovery (FLAIR) (TR/TE = 9,000/119 ms, inversion time 2,609 ms) images. Lower leg imaging was carried out in axial [field of view (FOV) 24-32 cm, slice thickness 10 mm, and slice gap 0.5-1.0 mm] and coronal planes (FOV 38-40 cm, slice thickness 4-5 mm, slice gap 0.5-1.0 mm).

### Histopathological studies

Histopathological analysis of the distal sural nerve was performed in a patient (II-2). The density of myelinated fibers (MFs), axonal diameter, and myelin thickness were determined directly from the semi-thin transverse sections using a computer-assisted image analyzer (AnalySIS, Soft Imaging System, Germany). Ultrathin cut samples (60 ~ 65 nm) are contrasted with uranyl acetate and lead citrate for ultrastructural study (H-7650, Hitachi, Japan).

### DNA preparation and whole exome sequencing

Total DNA was purified from peripheral blood using QIAamp blood DNA purification kit (QIAGEN, Hilden, Germany). DNAs were prescreened for duplication of 17p12 (*PMP22*) and mutations in the coding exons of *GJB1, MPZ, NEFL* and *MFN2* as previously described [9]. Exome sequencing and subsequent filtering was performed as previously described [9].

### Determination of exon5 splicing and DNA cloning

Total mRNA was purified from proband’s fibroblast using RNeasy minikit (QIAGEN). Then cDNA was synthesized using Superscript reverse transcriptase (Invitrogen, Carlsbad, CA). For amplification of wildtype and mutant HADHB, following primers are used: HADHB forward, 5′-ACG TCA GCC AAG ATT CCA GA-3′, and HADHB reverse, 5′-GCA CAG AAA CTT CAG GTC ACT TC-3′. Sequencing was performed to determine cDNA sequence of each amplified mutant allele. Amplified cDNAs were cloned into pCR2.1-TOPO vector (Invitrogen) and subsequently moved into expression vector, pCMV-myc (Clontech, Mountain View, CA).

### DNA transfection and determination of protein half-life

HEK293 cells (2 × 10^5^) were transfected with control vector, pCMV-myc, and cloned HADHB cDNAs using FuGene transfection reagent (Promega, Madison, WI). For determination of half-life of HADHB, cyclohexamide (100 μM) was treated after 20 hr of transfection.

### Western blotting

Protein expression in fibroblast and HEK293 cells were determined by standard Western blotting. Anti-HADHB Ab (Santa Cruz Biotechnology, Santa Cruz, CA), anti-myc Ab (Abcam, Cambridge, UK), anti-actin Ab, anti-mouse secondary Ab, anti-rabbit secondary Ab (Sigma, St. Louis, MO), and ECL plus Western blotting substrate (Thermo Scientific, Rockford, IL) were used for detection of protein.

## Results

### Identification of a compound heterozygous mutation in HADHB

To identify the underlying genetic cause of this family, WES was performed for five members (I-1, I-2, II-1, II-2, and II-4). Total sequencing yields was 10.11Gbp/sample, and mappable reads were 93.1%. Total number of variants (SNPs and indels) was 80,301 single nucleotide variants (SNVs)/sample, of which 20,430 SNVs were coding variants (Additional file 1: Table S1). Several functionally significant variants were identified in the CMT-relevant genes; however, no variant was fitted for autosomal recessive inheritance of the family (Additional file 1: Table S2). Moreover, most of them were also observed in controls.

**Figure 1:**
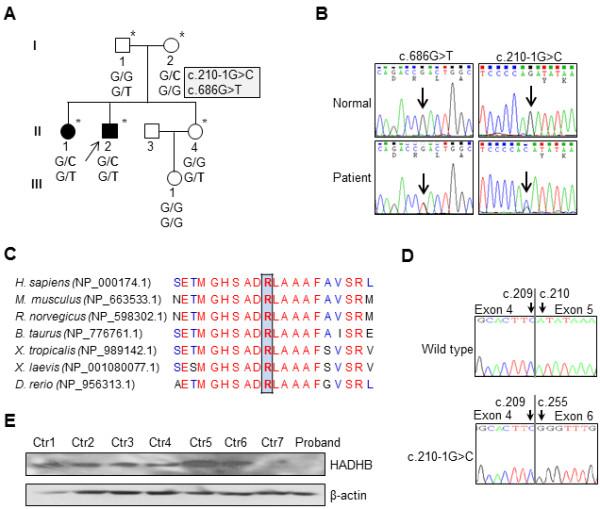
**Pedigree and *HADHB *mutations in FC354. (A)** Alleles of two *HADHB* mutations were indicated. Open symbols, unaffected; filled symbols, affected; arrow, proband; asterisks, individuals underwent WES. **(B)** Sequencing chromatograms of c.686G > T and c.210-1G > C mutation. Vertical arrows indicate the mutation site. **(C)** Conservation analysis of amino acid sequences among species. Complete conserved amino acids, red; partial conserved amino acids, blue. **(D)** Deletion of exon5 in c.210-1G > C allele was confirmed by cDNA sequencing. **(E)** Expression level of *HADHB* in the proband’s fibroblast. Ctr1, normal control fibroblast; and Ctr2-7, fibroblast from CMT patients with mutation in other than HADHB gene.

From the unreported or rare functionally significant SNVs reported in dbSNP135 and 1000 Genome project database, we could identify two pairs of compound heterozygous mutations transmitted from each of the parents: [c.210-1G > C] + [c.686G > T] in HADHB (NM_000183) and [c.C935T] + [c.697_698insTT] in Cyclin-dependent kinase-like 4 (CDKL4, NM_001009565). Since the CDKL4 compound mutations were found in Korean healthy controls, the HADHB compound heterozygous mutation was finally considered as the underlying cause of the axonal CMT (Figures 1A and B). The p.Arg229Leu mutation site locating in the thiolase N domain is highly conserved between different species (Figure 1C) and is predicted to affect the functional integrity by *in silico* analysis (SIFT and PolyPhen2). The other mutation, c.210-1G > C, is predicted and experimentally proved to result in the loss of exon 5 by improper splicing (Figure 1D).

Western blotting revealed that expression level of HADHB from the proband’s fibroblast is quite lower than those from controls (Figure 1E). Additionally, Western blotting revealed that the mutant proteins expressed in HEK293 cells are more unstable than wild-type (Additional file 2: Figure S1). Cyclohexamide treatment demonstrated that the half-life of wild-type *HADHB* is quite short (less than 2 hr). Thus these results are consistent with the lowered level of *HADHB* in patients.

### Clinical manifestations

The proband (Figure 1A, II-2) was born from non-consanguineous Korean parents. He noticed lower leg weakness, which resulted in bilateral foot drop and gait disturbance at 5 years. At 12 years old, he used a walker due to progressively impaired gait. Neurologic examination at 34 years of age revealed that muscle weakness and atrophy began and the distal part of the lower limbs was prominently involved. Toe and heel gait defects were found. Vibration and position senses were more severely disturbed than pain and temperature senses. Deep tendon reflexes were absent in all extremities, but pathologic reflexes were not found. An echocardiogram showed normal and there was no evidence of intermittent rhabdomyolysis or retinal dysfunction. Ophthalmological examination including dilated fundus examination was done in both patients, but pigmentary retinopathy was not observed. Laboratory findings showed a total carnitine level of 33.4 μmol/L (reference interval: 45-91 μmol/L), a free carnitine level of 24.5 μmol/L (reference interval: 36-74 μmol/L), and acylcarnitine level of 10.9 μmol/L (reference interval: 36-74 μmol/L).

His 37-year-old elder sister (II-1) began with distal limb weakness and gait disability from 6 years old and became to use a walker from 15 years old. At 23 years, she underwent an operation of bilateral feet due to walking difficulties. When we examined her at 37 years, she also revealed muscle weakness and atrophies of bilateral distal muscles of the upper and lower limbs. Loss of sensory and tendon reflexes were similar to the proband. The other family members were proven normal by careful clinical and electrophysiological examination.

The nerve conduction studies (NCSs) revealed that motor nerve conduction velocities (MNCVs) of median, ulnar, peroneal and tibial nerves in both patients were abnormal (Table 1). Except median nerve of II-1, all compound muscle action potentials (CMAPs) were below the normal range. Sensory nerve action potentials (SNAPs) of all tested nerves were lost. Needle electromyography (EMG) was compatible with neuropathy with fibrillation potentials and neurogenic motor unit action potentials (MUAPs).

**Table 1:** **Electrophysiological features of patients with mutations in *HADHB *** **gene**

**Patients**	**II-1**	**II-2**		**Normal value**
Age at exam (years)	37	32	34	
Median nerve				
TL (ms)	**4.2**	**4.1**	**4.1**	< 3.9
CMAP (mV)	7.2	**2.2**	**3.6**	> 6.0
MNCV (m/s)	**44.9**	**40.6**	**41.9**	> 50.5
F-wave (ms)	**36.8**	**55.0**	**38.8**	< 28.0
Ulnar nerve				
TL (ms)	**3.5**	**4.0**	**4.0**	< 3.0
CMAP (mV)	**5.7**	**5.2**	**5.8**	> 8.0
MNCV (m/s)	**40.4**	**43.1**	**43.9**	> 51.1
F-wave (ms)	**33.2**	**36.8**	**34.4**	< 29.0
Peroneal nerve				
TL (ms)	**A**	**A**	**A**	< 5.3
CMAP (mV)	**A**	**A**	**A**	> 1.6
MNCV (m/s)	**A**	**A**	**A**	> 41.2
F-wave (ms)	**A**	**A**	**A**	< 49.0
Tibial nerve				
TL (ms)	**3.9**	**A**	**A**	< 5.4
CMAP (mV)	**0.2**	**A**	**A**	> 6.0
MNCV (m/s)	**34.0**	**A**	**A**	> 41.1
F-wave (ms)	**63.0**	**A**	**A**	< 52.1
Median sensory nerve				
SNAP (μV)	**A**	**A**	**A**	> 8.8
SNCV (m/s)	**A**	**A**	**A**	> 39.3
Ulnar sensory nerve				
SNAP (μV)	**A**	**A**	**A**	> 7.9
SNCV (m/s)	**A**	**A**	**A**	> 37.5
Sural nerve				
SNAP (μV)	**A**	**A**	**A**	> 6.0
SNCV (m/s)	**A**	**A**	**A**	> 32.1

Bold character indicates abnormal values. A, absent potentials; TL, terminal latency; CMAP, compound muscle action potential; MNCV, motor nerve conduction velocity; SNAP, sensory nerve action potential; SNCV, sensory nerve conduction velocity; NP, no potential.

### Histopathological findings

**Figure 2:**
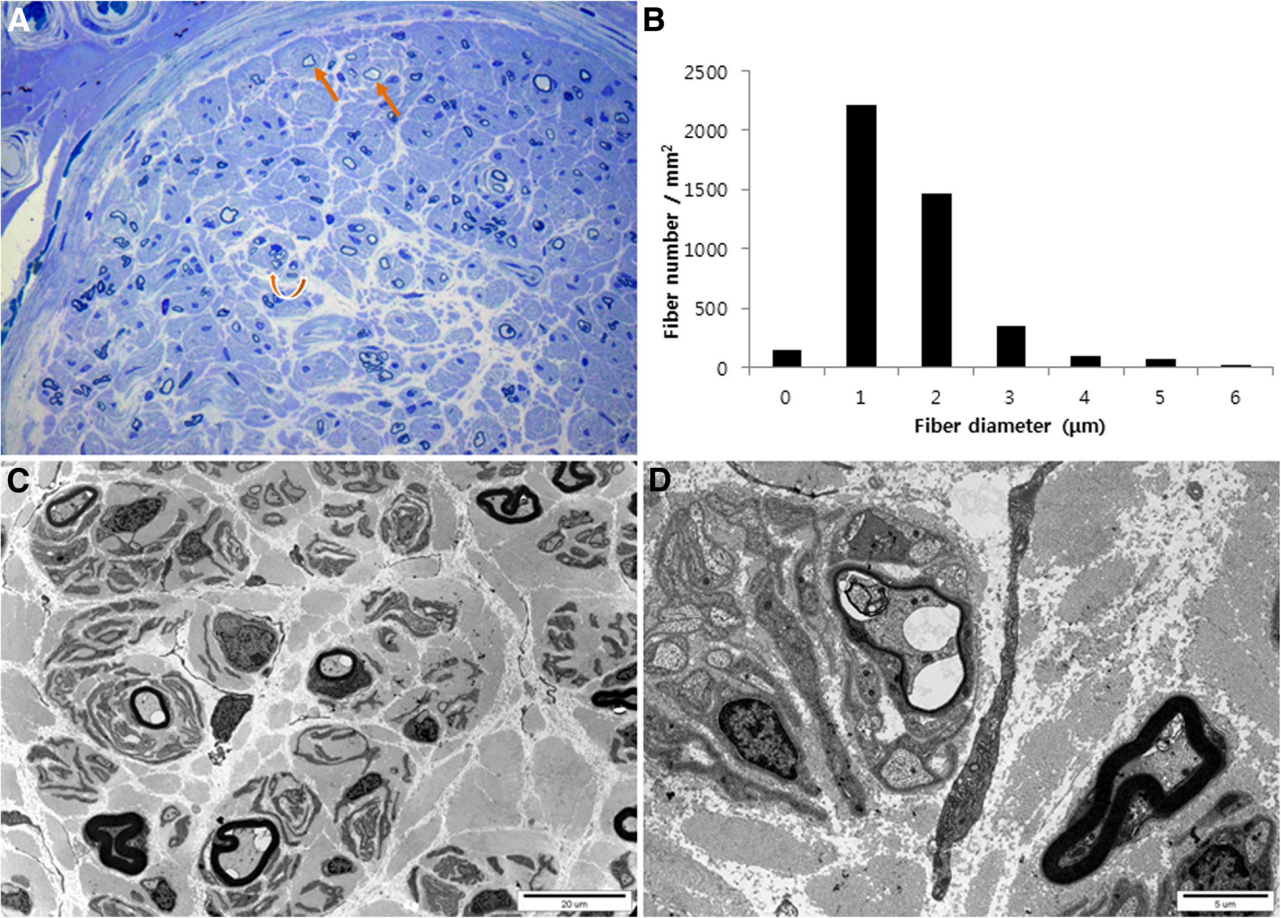
**Histopathological findings of distal sural nerve biopsy in II-2. (A)** Toluidine blue-stained semi-thin transverse section revealed absence of large MFs and remaining medium and small-sized MFs with frequently noted thin MFs (arrows) and occasionally noted axonal clusters (curved arrow). **(B)** Histogram showed unimodal distribution pattern of MF diameter. **(C and D)** Electron micrographs revealed MFs with pseudo-onion bulb formation and a thin MF compatible with remyelination. Original magnifications: **A**, x400; **C**, x3000; and **D**, x10000.

Histopathological analysis of the distal sural nerve was performed in the proband at 34 years of age. Semi-thin transverse sections with toluidine blue stain showed absence of large myelinated fibers (MFs) with remaining medium and small-sized MFs, and occasionally noted regenerating axonal clusters (Figure 2A). Thin MFs were frequently noted and remaining MFs (4,366/mm^2^) were less than control (45-year-old male: 7,300/mm^2^). The average of diameter of MFs and MF% area were also quite lower than control (Figure 2B). Electron microscopic examination revealed MFs with pseudo-onion bulb formation, occasionally noted regenerating clusters, and rarely noted demyelinated axon and thin MFs (Figures 2C and D). Several myelinated and unmyelinated axons showed swelling or vacuolization of axoplasm, swollen or abnormal mitochondria, and abnormal membranous structures.

### Lower limb MRI findings

**Figure 3:**
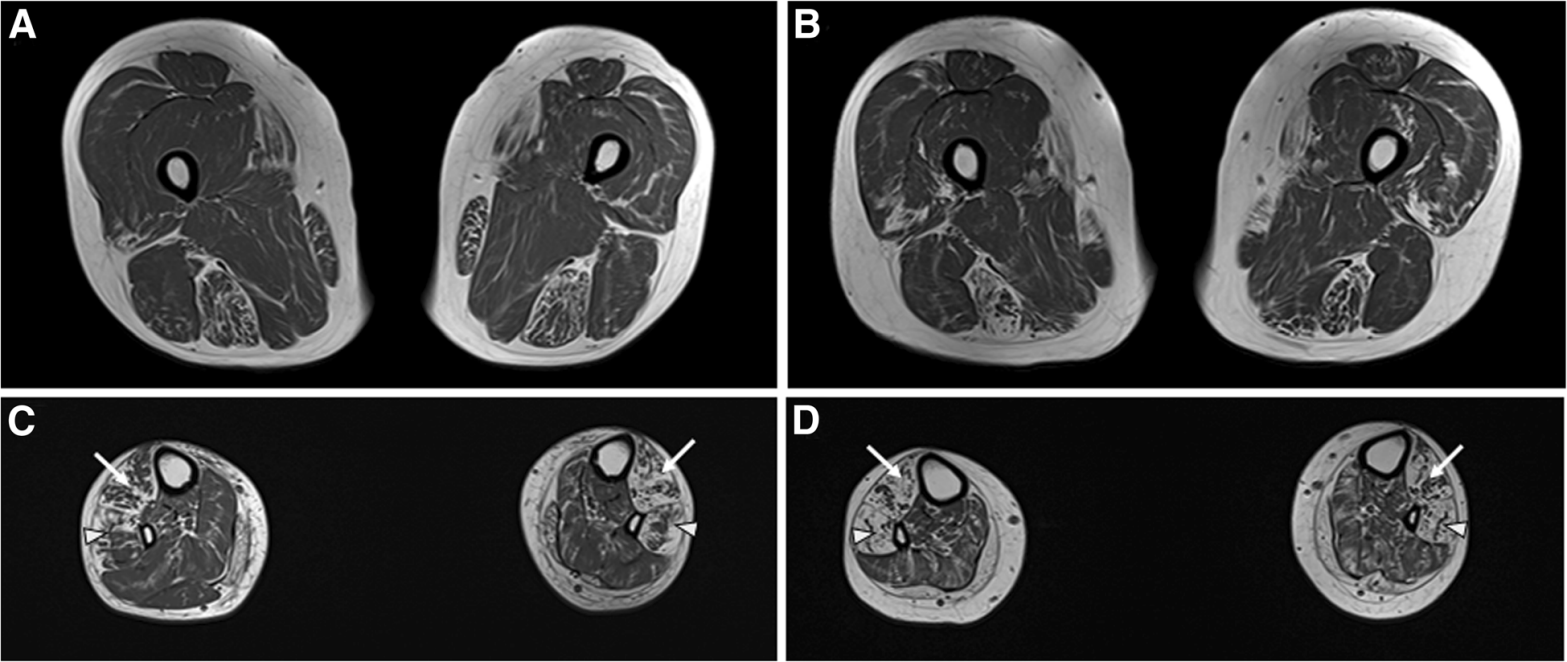
**Thigh and lower leg MRIs in II-1 (A and C) and II-2 (B and D). (A and B)** At the thigh level, MRIs demonstrated severe muscle atrophied with signal changes of semitendinous muscles of posterior compartment, gracilis muscle of medial compartment, and sartorius muscle of anterior compartment. **(C and D)** The lower leg MRIs revealed selective severe muscle atrophy with signal change of anterior (arrow; tibialis anterior, extensor digitorum and halluces longus muscles) and lateral (arrowhead; peronei muscles) compartments. However, posterior compartment muscles (soleus and tibialis posterior muscles) were sparing in the later stages.

Brain and pelvic MRIs showed normal findings (data not shown), but lower limb MRIs of both patients revealed hyperintense signal abnormalities (Figure 3). T1-weighted images demonstrated selective muscle atrophies with signal changes, which were more prominent in lower leg muscles than thigh or pelvic muscles, thereby being consistent with the hypothesis of length-dependent axonal neuropathy. At the thigh level, there were severe involvements of the semitendinosus, sartorius and gracilis muscles, but a sparing of vastus group and adductor muscles. The lower leg MRI revealed marked involvement of tibialis anterior, peronei, extensor digitorum and halluces longus muscles; however, soleus muscles were sparing.

## Discussion

Mitochondrial trifunctional protein (MTP) exerts a significant catalytic activity for last three steps of long chain acyl-CoA fatty acids and consists of heterooctamer of α-subunit (*HADHA*) and β-subunit (*HADHB*) [10]. To date, 33 mutation sites in *HADHB* gene have been reported [17] and these mutation-associated phenotypes exhibit clinical heterogeneity, which can be mainly subdivided into three distinctive groups: severe neonatal presentation with cardiomyopathy, hepatic form with recurrent hypoketotic hypoglycemia, and later-onset axonal sensory neuropathy with episodic myoglobinuria [11].

Several research groups have attempted to elucidate the underlying genotype-phenotype correlation of the *HADHB* gene. Spiekerkoetter et al. analyzed mutations from 15 patients and linked clinical phenotype with the location of the mutation [18]. They postulated that mutations in the outer loop were present only in milder forms. In addition, they suggested that the degree of reduction of cross-reactive immunological material (CRIM) is also correlated with the severity of clinical presentation. Purevsuren et al. also demonstrated the positive correlations between residual enzymatic activity and phenotypic severity using *in vitro* functional study [19]. Collectively, although MTP deficiency is recessive and highly heterogeneous, there are strong correlations between phenotypic severity and residual enzymatic activity or CRIM amounts of mutant proteins.

According to the crystal structure of HADHB, present mutation, Arg229Leu, locates in the outer loop [20]. This implicates that Arg229Leu mutation might mildly affect LCKT activity, while splicing variant of C.210-1G > C, which results in deletion of 15 amino acids, was predicted to have severely affected LCKT activity. In contrast to these predictions, however, overexpression of these mutants revealed that mutant proteins primarily possess lower stability than wild type. These results might come from the conformation changes of their structures, thereby translated misfolded proteins are subsequently degraded by proteases. Therefore, the loss of the enzymatic activity by the mutations might be greater than predicted.

To unveil the effect of *HADHB* deficiency on peripheral nerve, we first analyzed the expression of *HADHB* in distal sural nerve. Transcriptome analysis from 14 control samples confirmed the expression of *HADHB* in the distal sural nerve (data not shown). In addition, we tried to demonstrate the effect of *HADHB* deficiency on peripheral nerve; however, the effects of *HADHB* knockdown on the responses to oxidative stress and cell proliferation of mouse motor neuron were not within statistically significant ranges (data not shown). Therefore, further analyses are needed to elucidate the pathophysiological mechanism of *HADHB* mutation-mediated peripheral neuropathy.

Clinical and histopathological features of the present patients were consistent with an early-onset axonal sensorimotor CMT neuropathy. Both patients revealed the age at onset below 10 years old, and showed typical clinical phenotypes of CMT including predominant distal muscle weakness and atrophy, pes cavus, and steppage gait (Additional file 3: Figure S2). Nerve conduction studies in both of them were compatible with motor and sensory neuropathies. However, they did not have any other previously reported symptoms of *HADHB* patients, such as a rhabdomyolysis, cardiomyopathy, hypoketotic hypoglycemia, metabolic encephalopathy, liver dysfunction, nor pigmentary retinopathy [11]. Moreover, distal sural nerve biopsy in II-2 patient revealed typical axonal neuropathy. It is noteworthy that swollen or abnormal mitochondria were noted in several MFs and unmyelinated axons, of which significance was uncertain. Previously loss of MFs with features of Wallerian degeneration [21] and axonal and myelin degeneration [18] were reported in *HADHB* patients. MRI analysis indicated a muscular involvement in the present patients with *HADHB* mutation. In addition, lower limb MRI findings revealed severe muscle atrophy with hyperintense signal changes in the lower leg muscles than those in the thigh or hip muscles, which was compatible with the typical features of the length-dependent axonal degeneration.

## Conclusion

In conclusion, we first report that a heterozygous mutation in *HADHB* causes early-onset axonal CMT without any typical clinical symptom of MTP based on the data from WES, MRI, and histopathological and electrophysiological analysis. Thus our findings suggest that *HADHB* gene can be also filed as a causative of CMT, which expands the clinical spectrum of both *HADHB* related disease and hereditary motor and sensory neuropathy.

## Competing interests

The authors declare that they have no competing interests.

## Authors’ contributions

Study concept and design: CKW, and CB-O. Acquisition of data: HYB, LJH, PJ-M, CY-R, HYS, YBR, YJH, and KH. Analysis and interpretation of data: HYB, YJH, KH, PJ-M, CS-C, CKW, and CB-O. Drafting of the manuscript: HYB, CKW, and CB-O. Critical revision of the manuscript for important intellectual content: JS-C. Obtained funding: CKW, and CB-O. Administrative, technical, and material support: LJH, CY-R, HYS, YBR, and PJ-M. Study supervision: CKW and CB-O. All authors read and approved the final manuscript.

## Pre-publication history

The pre-publication history for this paper can be accessed here:

http://www.biomedcentral.com/1471-2350/14/125/prepub

## Supplementary Material

Additional file 1: Table S1 Summary of exome sequencing data for 5 samples. **Table S2.** Polymorphic functionally significant variants in CMT-relevant genes. Click here for file

Additional file 2: Figure S1 **(A)** Western blotting of wild-type (WT), c.686G > T and c.210-1G > C mutants expressed in HEK293 cells. Cyclohexamide (CHX) was treated for indicated time. First lane, cells transfected with pCMV-myc control vector. **(B)** Western blotting of proband’s fibroblast after treatment of CHX. Ctr5-7, fibroblast from CMT patients with mutation in other than HADHB gene. Click here for file

Additional file 3: Figure S2 Leg pictures of the patient (II-2). The patient showed prominent distal muscle atrophies of both lower extremities and revealed typical CMT phenotypes of pes cavus, and steppage gait. Click here for file
