# Supplementary material for: A compound heterozygous mutation in HADHB gene causes an axonal Charcot-Marie-tooth disease
Source: BMC Med Genet. 2013 Dec 5;14:125. doi: 10.1186/1471-2350-14-125 (PMC4029087; doi:10.1186/1471-2350-14-125)
Supplement: Additional file 1: Table S1 — Summary of exome sequencing data for 5 samples. Table S2. Polymorphic functionally significant variants in CMT-relevant genes. [file 1471-2350-14-125-S1.docx]

Additional file 1

**Table S1** Summary of exome sequencing data for 5 samples.

| Samples | Affected | | |  | | Unaffected | | |
| --- | --- | --- | --- | --- | --- | --- | --- | --- |
|  | II-1 | II-2 |  | | I-1 | | I -2 | II-4 |
| Total yields (Gbp) | 6,44 | 6,71 |  | | 15,68 | | 8,47 | 13,27 |
| Mappable reads (%) | 94.00 | 92.90 |  | | 92.90 | | 92.70 | 93.20 |
| On-target reads (%) | 70.00 | 76.20 |  | | 37.10 | | 38.30 | 39.40 |
| Coverage of target regions (≥ 1X) | 97.40% | 97.3% |  | | 95.60% | | 95.20% | 96.00% |
| Coverage of target regions (≥ 10X) | 91.60% | 91.8% |  | | 91.30% | | 85.50% | 91.60% |
| Mean read depth of target regions | 81.3 | 93.3 |  | | 59.8 | | 34.8 | 49.9 |
| Total number of SNPs | 49,839 | 54,317 |  | | 87,514 | | 82,060 | 86,998 |
| Number of coding SNPs | 18,871 | 19,089 |  | | 20,606 | | 20,580 | 20,714 |
| Total number of indels | 8,616 | 9,118 |  | | 7,947 | | 7,006 | 8,088 |
| Number of coding Indels | 484 | 514 |  | | 424 | | 434 | 434 |
| Candidate CMT gene | 0 | | | | | | | |
| Candidate gene | 1 | | | | | | | |

^a^Nonsynonymous variants include splicing site, frameshift, stop-gain, and stop-loss.

**Table S2** Polymorphic functionally significant variants in CMT-relevant genes

| Gene | RefSeq^a^ | Chr:position | Variants | | dbSNP135^c^ | 1000G^c^ | Affected | | Unaffected | | | Remark^d^ |
| --- | --- | --- | --- | --- | --- | --- | --- | --- | --- | --- | --- | --- |
|  |  |  | Nt^b^ | AA |  |  | II -1 | II -2 | I-1 | I -2 | II-3 |  |
| *ARHGEF10* | NM_014629.2 | chr8: 1833801 | c.1110G>C | L370F | rs9657362 | 0.14 | GC | GC | GC | GG | GC | NC |
|  |  | chr8: 1824881 | c.824G>A | R275H | rs145821459 | < 0.01 | GG | GA | GG | GA | GA |  |
| *CTDP1* | NM_004715.4 | chr18: 77475309 | c.1849G>A | E617K | rs145888904 | NR | GA | GA | GA | GG | GA | NC |
|  |  | chr18: 77473127 | c.1019C>T | T340M | rs2279103 | 0.11 | CC | CT | CT | CT | CC |  |
| *DMPK* | NM_001081563.1 | chr19: 46275976 | c.1297C>G | L433V | rs527221 | 0.12 | CC | CG | CG | CC | CC | NC |
| *DNMT1* | NM_001379.2 | chr19: 10273372 | c.931A>G | I311V | rs2228612 | 0.18 | AA | AG | AG | AA | AA | NC |
|  |  | chr19: 10291113 | c.358G>C | V120L | rs75616428 | 0.01 | GG | GC | GG | GC | GG |  |
| *DYNC1H1* | NM_001376.4 | chr14: 102506647 | c.11765C>T | P3922L | rs141696238 | 0.01 | CT | CC | CC | CT | CT | NC |
|  |  | chr14: 102509084 | c.12512A>G | K4171R | NR | NR | AG | AA | AG | AA | AG |  |
| *FIG4* | NM_014845.5 | chr6: 110064928 | c.1090A>T | M364L | rs2295837 | 0.10 | AT | AA | AT | AA | AT | NC |
|  |  | chr6: 110107517 | c.1961T>C | V654A | rs9885672 | 0.37 | TC | TT | TC | TT | TC |  |
| *GARS* | NM_002047.2 | chr7: 30634661 | c.124C>G | P42A | rs1049402 | 0.67 | CG | CG | CG | CC | CG | NC |
| *IGHMBP2* | NM_002180.2 | chr11: 68678962 | c.602T>C | L201S | rs560096 | 0.70 | TT | TC | TT | TC | TC | NC |
|  |  | chr11: 68705674 | c.2636C>A | T879K | rs17612126 | 0.23 | CC | CA | CC | CA | CA |  |
| *IKBKAP* | NM_003640.3 | chr9: 111641825 | c.3473C>T | P1158L | rs1538660 | 0.22 | CT | CT | CT | CT | CT | NC |
|  |  | chr9: 111651620 | c.3214T>A | C1072S | rs3204145 | 0.22 | TA | TA | TA | TA | TA |  |
| *INF2* | NM_001031714.3 | chr14: 105173653 | c.1049C>T | P350L | rs146529868 | 0.01 | CT | CC | CT | CC | CT | NC |
| *LRSAM1* | NM_138361.4 | chr9: 130242166 | c.952A>G | N318D | rs1539567 | 0.74 | AG | AG | AG | AG | AG | NC |
| *NEFL* | NM_006158.3 | chr8: 24811071 | c.1413delC | P471fs | rs11300136 | NR | DD | DD | DD | DD | DD | NC |
| *PRX* | NM_181882.2 | chr19: 40900865 | c.3394G>A | G1132R | rs268674 | 0.96 | GA | GA | GA | GA | GA | NC |
| *SBF2* | NM_030962.3 | chr11: 9853777 | c.3646C>G | Q1216E | rs12574508 | 0.10 | CG | CG | CG | CC | CG | NC |
| *SEPT9* | NM_001113495.1 | chr17: 75494705 | c.1390A>G | M464V | rs2627223 | 0.92 | AG | AG | AG | AG | AG | NC |
| *SETX* | NM_015046.5 | chr9: 135139901 | c.7759A>G | I2587V | rs1056899 | 0.51 | AG | AG | AG | AG | AG | NC |
|  |  | chr9: 135173685 | c.5563A>G | T1855A | rs2296871 | 0.41 | AG | AG | AG | AG | AG |  |
|  |  | chr9: 135203409 | c.3576T>G | D1192E | rs1185193 | 0.66 | TG | TG | TG | TT | TG |  |
|  |  | chr9: 135202829 | c.4156A>G | I1386V | rs543573 | 0.59 | AG | AG | AG | AA | AA |  |
| *WNK1* | NM_213655.4 | chr12: 990912 | c.3922A>C | T1308P | rs956868 | 0.85 | AC | AC | AC | AA | AC | NC |
|  |  | chr12: 994487 | c.5273G>C | C1758S | rs7955371 | 0.99 | GC | GC | GC | GC | GC |  |
|  |  | chr12: 1005486 | c.6589C>T | R2197C | rs117016551 | < 0.01 | CC | CT | CT | CC | CT |  |

^a^GenBank registration number of reference sequence.

^b^cDNA numbering was achieved with +1, corresponding to the A of the ATG initiation codon.

^c^Registration number (dbSNP) or mutant allele frequency (1000 Genome Database), NR: non-reported.

^d^NC: non-cosegregated with affected individuals.
