# Supplementary figures and images for: A compound heterozygous mutation in HADHB gene causes an axonal Charcot-Marie-tooth disease
Source: BMC Med Genet. 2013 Dec 5;14:125. doi: 10.1186/1471-2350-14-125 (PMC4029087; doi:10.1186/1471-2350-14-125)

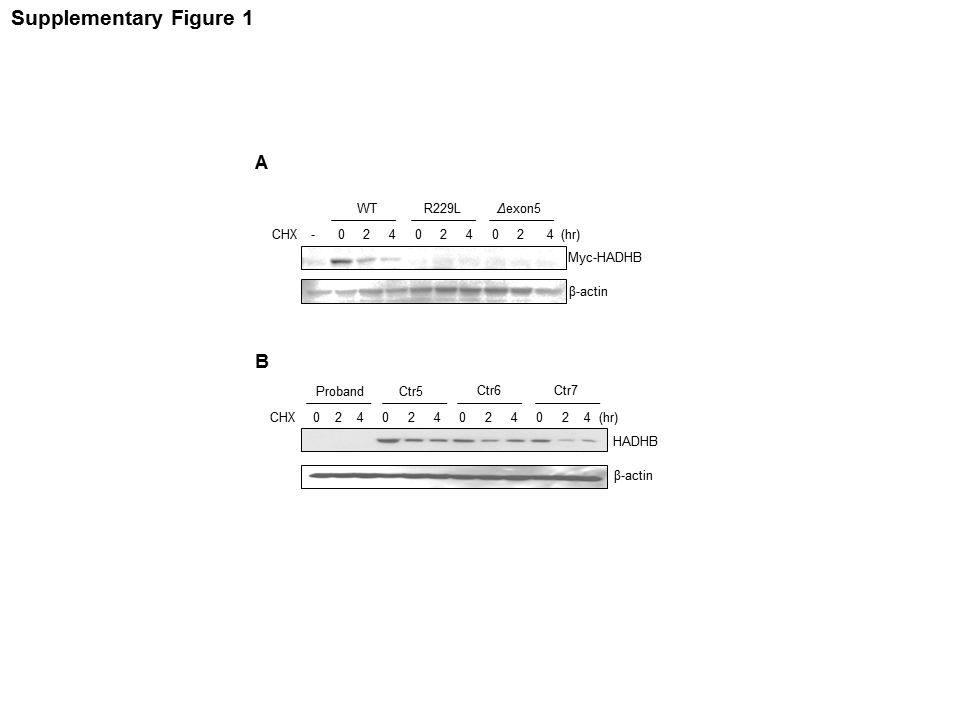

Supplement: Additional file 2: Figure S1 — (A) Western blotting of wild-type (WT), c.686G > T and c.210-1G > C mutants expressed in HEK293 cells. Cyclohexamide (CHX) was treated for indicated time. First lane, cells transfected with pCMV-myc control vector. (B) Western blotting of proband’s fibroblast after treatment of CHX. Ctr5-7, fibroblast from CMT patients with mutation in other than HADHB gene. [file 1471-2350-14-125-S2.tiff]

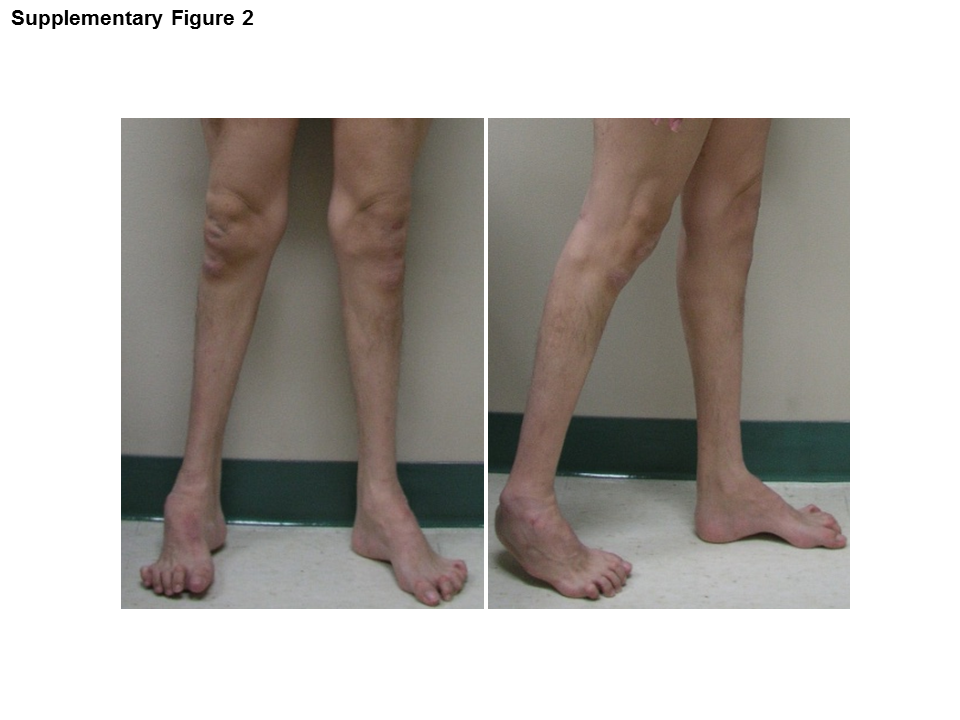

Supplement: Additional file 3: Figure S2 — Leg pictures of the patient (II-2). The patient showed prominent distal muscle atrophies of both lower extremities and revealed typical CMT phenotypes of pes cavus, and steppage gait. [file 1471-2350-14-125-S3.tiff]
